# Engineering recombinant replication-competent bluetongue viruses expressing reporter genes for *in vitro* and non-invasive *in vivo* studies

**DOI:** 10.1128/spectrum.02493-23

**Published:** 2024-02-14

**Authors:** Sergio Utrilla-Trigo, Luis Jiménez-Cabello, Alejandro Marín-López, Miguel Illescas-Amo, Germán Andrés, Eva Calvo-Pinilla, Gema Lorenzo, Piet A. van Rijn, Javier Ortego, Aitor Nogales

**Affiliations:** 1 Centro de Investigación en Sanidad Animal (CISA), Instituto Nacional de Investigación y Tecnología Agraria y Alimentaria (INIA), Valdeolmos, Madrid, Spain; 2 Section of Infectious Diseases, Department of Internal Medicine, Yale University School of Medicine, New Haven, Connecticut, USA; 3 Department of Virology, Wageningen Bioveterinary Research (WBVR), Lelystad, the Netherlands; 4 Department of Biochemistry, Centre for Human Metabolomics, North-West University, Potchefstroom, South Africa; Institute of Microbiology Chinese Academy of Sciences, Beijing, China

**Keywords:** bluetongue virus (BTV), non-structural protein 1 (NS1), reverse genetics, reporter gene, luciferase, fluorescent protein, IVIS

## Abstract

Bluetongue virus (BTV) is the causative agent of the important livestock disease bluetongue (BT), which is transmitted via Culicoides bites. BT causes severe economic losses associated with its considerable impact on health and trade of animals. By reverse genetics, we have designed and rescued reporter-expressing recombinant (r)BTV expressing NanoLuc luciferase (NLuc) or Venus fluorescent protein. To generate these viruses, we custom synthesized a modified viral segment 5 encoding NS1 protein with the reporter genes located downstream and linked by the Porcine teschovirus-1 (PTV-1) 2A autoproteolytic cleavage site. Therefore, fluorescent signal or luciferase activity is only detected after virus replication and expression of non-structural proteins. Fluorescence or luminescence signals were detected in cells infected with rBTV/Venus or rBTV/NLuc, respectively. Moreover, the marking of NS2 protein confirmed that reporter genes were only expressed in BTV-infected cells. Growth kinetics of rBTV/NLuc and rBTV/Venus in Vero cells showed replication rates similar to those of wild-type and rBTV. Infectivity studies of these recombinant viruses in IFNAR(−/−) mice showed a higher lethal dose for rBTV/NLuc and rBTV/Venus than for rBTV indicating that viruses expressing the reporter genes are attenuated *in vivo*. Interestingly, luciferase activity was detected in the plasma of viraemic mice infected with rBTV/NLuc. Furthermore, luciferase activity quantitatively correlated with RNAemia levels of infected mice throughout the infection. In addition, we have investigated the *in vivo* replication and dissemination of BTV in IFNAR (−/−) mice using BTV/NLuc and non-invasive *in vivo* imaging systems.

**IMPORTANCE:**

The use of replication-competent viruses that encode a traceable fluorescent or luciferase reporter protein has significantly contributed to the *in vitro* and *in vivo* study of viral infections and the development of novel therapeutic approaches. In this work, we have generated rBTV that express fluorescent or luminescence proteins to track BTV infection both *in vitro* and *in vivo*. Despite the availability of vaccines, BTV and other related orbivirus are still associated with a significant impact on animal health and have important economic consequences worldwide. Our studies may contribute to the advance in orbivirus research and pave the way for the rapid development of new treatments, including vaccines.

## INTRODUCTION

Bluetongue (BT) is a hemorrhagic disease of wild and domestic ruminants that produce a highly variable mortality rate and a significant worldwide economic impact (1–3). This World Organization for Animal Health notifiable disease is caused by Bluetongue virus (BTV), included in the *Orbivirus* genus within the family *Sedoreoviridae*, and transmitted by biting midges of the *Culicoides* genus (4). BTV is composed of an icosahedral capsid (~90 nm in diameter) where 3 concentric protein layers protect a 10 double-stranded RNA (dsRNA) segmented genome that encodes for 7 structural proteins (VP1, VP2, VP3, VP4, VP5, VP6, and VP7) and 5 non-structural proteins (NS1, NS2, NS3/NS3A, NS4, and the putative protein NS5) (5, 6). VP2 protein is involved in virus-entry and forms the outer capsid layer together with VP5 (7, 8). Moreover, VP2 is the main inductor of neutralizing antibodies and defines the serotype (9). To date, up to 29 serotypes of BTV have been described, which are classified as classical (BTV 1–24) or atypical (serotypes 25–29), and its distribution has increased considerably over the last decade (10–12). Vaccination against BTV constitutes the most effective prophylactic measure for BT control (13–15). However, current commercially available vaccines present several drawbacks, such as their serotype-specificity and novel vaccine approaches are highly needed.

Despite establishment of reverse genetics (RG) systems for orbiviruses has been challenging (16), successful development of RG technologies for these segmented dsRNA viruses has elicited a deeper understanding of diverse aspects of virus biology, pathogenesis as well as for vaccine development (17). In this sense, knowledge on BTV has benefited from the advent of the first RG system developed by Boyce & Roy in 2007 (18). Notwithstanding the technical complexity of this procedure, BTV core-derived full-length capped RNA transcripts were successfully used for recombinant (r)BTV recovery since viral cores are transcriptionally active (18). This RG strategy was optimized afterward by exclusively using *in vitro* synthesized T7 run-off transcripts derived from cDNA clones (19, 20). Further research proved that expression of the transcriptase complex (formed by VP1, VP4, and VP6), the major subcore protein VP3, and the non-structural proteins NS1 and NS2, prior to transfection with *in vitro* synthesized T7 transcripts of all 10 BTV segments, greatly improved the efficiency of rBTV recovery (21). Moreover, replacement of RNA transcripts by plasmid-driven expression during the first transfection enhanced rBTV rescue efficiency (22). mRNA or plasmid-driven initial protein synthesis followed by transfection of RNA transcripts have been extensively used for studies of infectivity (23–25), transmissibility (26, 27), pathogenicity (28), vector competence (29), identification of novel viral proteins, and characterization of protein function (5, 30–32). Moreover, these RG systems have facilitated the development of novel and DIVA (differentiating infected from vaccinated animals) (33–35) live-attenuated vaccines, such as DISA [Disabled Infectious Single Animal, by the deletion of the NS3/NS3a that blocks transmission to the insect vector (36)] or DISC [Disabled Infectious Single Cycle, based on the deletion of VP6 which impedes viral replication in inoculated animals (37, 38)] strategies. Recently, efficient PCR- or plasmid-based RG systems were developed to generate rBTV, resolving some RNA-based RG systems issues, like the heterogeneity of 3′ ends of the run-off transcripts, as well as they probably lengthen T7 transcripts half-life inside the cell, which could have positive implications on BTV recovery rate (39, 40).

Generation of replication-competent, reporter-expressing viruses by RG is a common and very useful molecular approach to study viral replication, identification of host factors, understanding viral pathogenesis, and very handy for high-throughput screening of antiviral drugs and neutralizing antibodies (41–45). Although there are many reporter genes with different properties, fluorescent and bioluminescent proteins are the preferred choice for researchers due to their high sensitivity and the continuous improvement of the technologies associated with their detection (44, 46–50). Recombinant viruses expressing these reporters not only permit *in vitro* characterization of viral infections but can also be used as a valid mean to track viral infection dynamics in different animal models (45, 51, 52).

In this work, using state-of-the-art plasmid-based RG methods, we have engineered a panel of rBTV viruses, representing the main BTV serotypes circulating in Europe (53–55), expressing fluorescent or luminescence reporter genes. Considering the lack of effective tools for enhancing research to explore the detailed genetic and molecular basis of BTV infection and pathogenesis (56), these viruses represent an excellent option to perform fundamental and applied research. Notably, our strategy could also be applied with other veterinary relevant orbivirus, enhancing global orbivirus research.

## RESULTS

### Generation of replication-competent rBTVs expressing reporter genes

**Fig 1:**
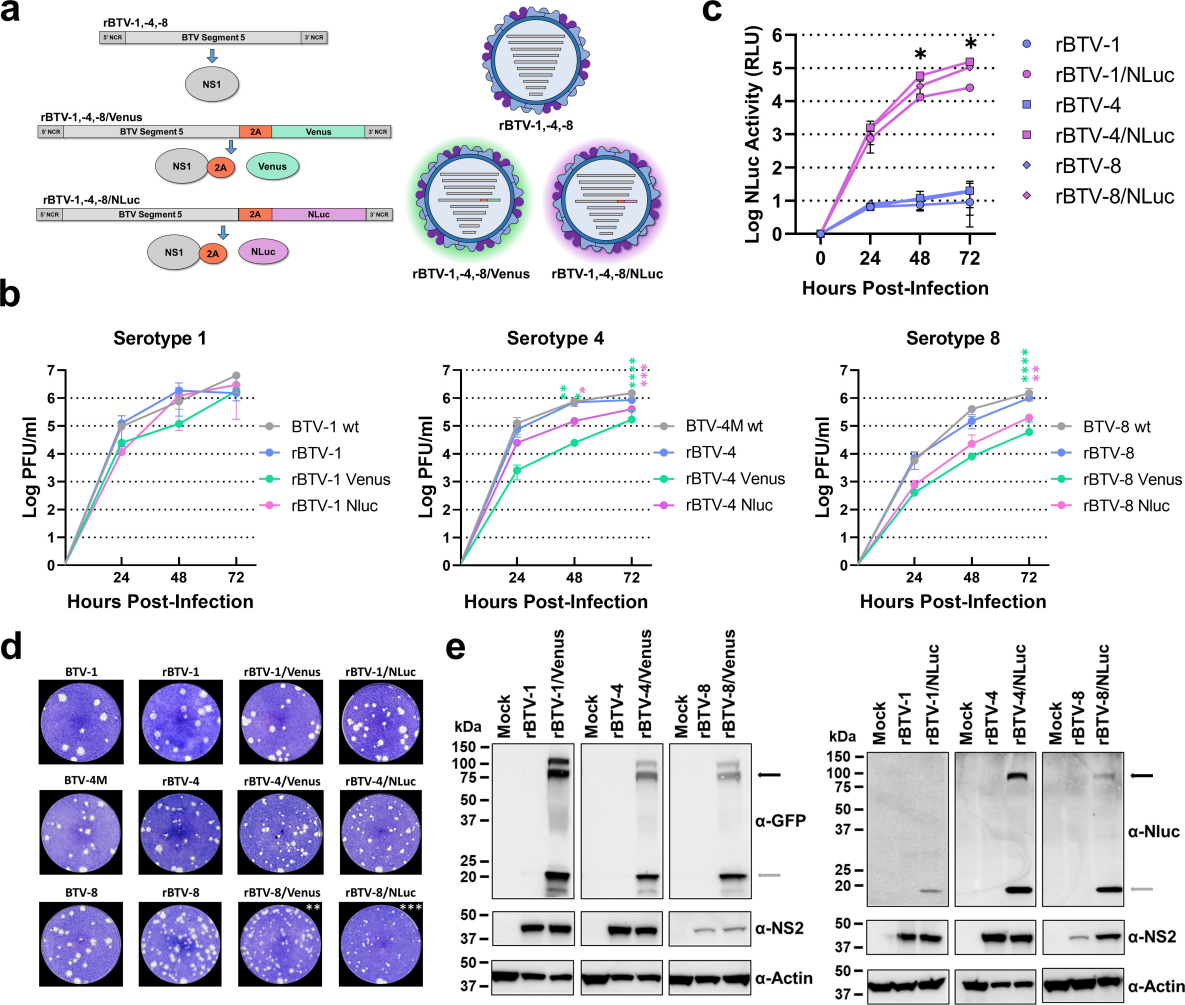
*In vitro* characterization of reporter-expressing rBTV. (**a**) Diagrammatic representation of the rescued recombinant viruses. Venus and NLuc reporter genes were included as fusion proteins by means of a 2A picornavirus peptide fused to the 3′ end of the segment 5 of BTV-1. (**b**) Viral replication kinetics of synthetic viruses in Vero cells. Confluent monolayers were infected with each BTV at an MOI of 0.01, and the virus supernatants were titrated as described in Materials and Methods assays were performed in triplicate (*n* = 3). Statistical differences between replication curves of rBTV-4 and rBTV-8 expressing Venus or NLuc and their corresponding wild-type viruses at different post-infection times were observed (Holm-Šídák method). *P < 0.05; **P < 0.0021; ***P < 0.0002; ****P < 0.0001. The error bars correspond to the standard deviation. (**c**) NLuc activity measured in tissue culture supernatants from cells infected with rBTV-1, rBTV-1/NLuc, rBTV-4, rBTV-4/NLuc, rBTV-8, or rBTV-8/NLuc. RLU: relative light units. Assays were performed in triplicate (*n* = 3). The error bars correspond to the standard deviation. (**d**) Plaque phenotype. Representative pictures of viral plaques in Vero cells (6-well-plate format) infected with the indicated viruses are shown. Virus lysis plaques stained with crystal violet. The area of 10 plaques was measured and statistics were performed comparing each of the rescued viruses with the WT by unpaired *t*-test (*P < 0.05). (**e**) Analysis of protein expression. Vero cells were mock infected or infected (MOI of 0.01) with rBTV-1, rBTV-1/NLuc, rBTV-1/Venus, rBTV-4, rBTV-4/NLuc, rBTV-4/Venus, rBTV-8, rBTV-8/NLuc, or rBTV-8/Venus. Protein expression was examined by Western blotting at 48 h.p.i using specific antibodies for GFP (Venus), NLuc, and NS2. Actin was used as a loading control. The numbers on the left indicate the molecular size of the protein markers in kilodaltons (kDa). The NS1-2A-reporter and the reporter (Nluc or Venus) proteins are indicated with black and gray arrows.

Generation of replication-competent reporter-expressing rBTVs requires that the inserted reporter gene does not affect key functions of the viral genome segment to which it is appended. In addition, the foreign sequences must not disrupt the packaging signals of the viral segments or affect other important regulatory RNA regions. Considering multiple factors such as the small–middle size of NS1 (about 550 amino acids), the high level of expression in host cells at early steps of infection, the previous knowledge about the viral NS1 functions and structure (57–59), alongside the experience in our laboratory with this viral protein (60–63), we attempted to modify the segment 5 of BTV in order to express a reporter gene that could serve to assess viral replication both *in vitro* and *in vivo*. To that end, we engineered a modified segment 5 containing a multicloning site to introduce the reporter sequence in frame with the NS1 gene (without the stop codon). Moreover, the porcine teschovirus-1 (PTV-1) 2A autoproteolytic cleavage site was introduced at the 3′ end followed by the multicloning site. Then, reporter genes were inserted into the multicloning site without altering the untranslated regions (UTRs) (Fig. 1a). As a result, NS1 and reporter proteins were co-translated.

To rescue rBTV, we combined two previously described transfection protocols (22, 39), where BSR-T7 cells were first co-transfected with six expression plasmids encoding the open reading frames (ORFs) of VP1, VP3, VP4, VP6, NS1, and NS2 of BTV6/net08 (22). Then, cells were co-transfected with different combinations of RG plasmids to generate the rBTV indicated in Table 1 (see also Table 2 for the sequences of BTV genes). Although the first transfection step is not required for rBTV recovery, we found that this step increases the success of BTV recovery (data not shown). Although in the first step of viral rescue (P0) some chimeric particles could be formed because the presence of proteins of BTV6/net08 in the cell, these chimeric particles will contain only the genome from the reverse genetic plasmids. Therefore, after this initial step, all viral particles will have the gene and protein constellation designed by the researchers since the BTV-6 genes could not be propagated. The recovery of rBTVs of serotypes 1, 4, and 8 of BTV was selected because of their active circulation in Europe (53–55). Before attempting to recover reporter-expressing rBTV, rescue of rBTV-1, rBTV-4, and rBTV-8 was confirmed to corroborate that all wild-type (WT) reverse genetic plasmids were functional. Next, we attempted the rescue of rBTV expressing two kinds of reporter proteins: Venus fluorescent protein, chosen because of the brightness and low toxicity in other RNA viruses (44, 64) and NanoLuc luciferase (NLuc), which was selected due to its small size (about 20 kDa), stability, and brightness (65, 66) (Fig. 1a; Table 1). Importantly, all reporter-expressing rBTV were recovered, which indicate the feasibility of our strategy to generate rBTV encoding heterologous genes from the viral segment 5.

**TABLE 1:** Recombinant viruses generated in this study

Virus	Genome constellation
rBTV-1	S1, S3, S4, S5, S7, S8, and S10 from BTV-4M S2, S6, and S9 from BTV-1
rBTV-1/Venus	S1, S3, S4, S5-Venus, S7, S8, and S10 from BTV-4M S2, S6, and S9 from BTV-1
rBTV-1/NLuc	S1, S3, S4, S5-NLuc, S7, S8, and S10 from BTV-4M S2, S6, and S9 from BTV-1
rBTV-4	S1, S3, S4, S5, S7, S8, and S10 from BTV-4M S9 from BTV-1 S2 and S6 from BTV-4
rBTV-4/Venus	S1, S3, S4, S5-Venus, S7, S8, and S10 from BTV-4M S9 from BTV-1 S2 and S6 from BTV-4
rBTV-4/NLuc	S1, S3, S4, S5-NLuc, S7, S8, and S10 from BTV-4M S9 from BTV-1 S2 and S6 from BTV-4
rBTV-8	S1, S3, S4, S5, S7, S8, and S10 from BTV-4M S9 from BTV-1 S2 and S6 from BTV-8
rBTV-8/Venus	S1, S3, S4, S5-Venus, S7, S8, and S10 from BTV-4M S9 from BTV-1 S2 and S6 from BTV-8
rBTV-8/NLuc	S1, S3, S4, S5-NLuc, S7, S8, and S10 from BTV-4M S9 from BTV-1 S2 and S6 from BTV-8

**TABLE 2:** BTV viral segments used in this study

Segment	BTV serotype	GenBank accession number
1	BTV-4M (MOR2009/09)	KP820944.1
2	BTV-1	FJ969720.1
2	BTV-4	AJ585125.1
2	BTV-8	AM498052.2
3	BTV-4M (MOR2009/09)	KP821186.1
4	BTV-4M (MOR2009/09)	KP821306.1
5	BTV-4M (MOR2009/09)	KP821426.1
6	BTV-1	FJ969723.1
6	BTV-4	AJ586699.1
6	BTV-8	AM498056.2
7	BTV-4M (MOR2009/09)	KP821668.1
8^*a*^	BTV-4M (MOR2009/09)	KP821788.1
9	BTV-1	FJ969727.1
10	BTV-4M (MOR2009/09)	KP822029.1

^*a*^ Two silent point mutations (T635C and G637C) were introduced in this viral segment.

### Characterization of rBTVs growth properties and plaque phenotype

We assessed virus fitness in cell culture by examining the multicycle growth properties (Fig. 1b and c) and plaque formation (Fig. 1d) of rBTV-1, rBTV-1/NLuc, rBTV-1/Venus, rBTV-4, rBTV-4/NLuc, rBTV-4/Venus, rBTV-8, rBTV-8/NLuc, and rBTV-8/Venus. WT BTV-1, BTV-4, and BTV-8 were included as controls. For virus growth kinetics, monolayers of Vero cells were infected (MOI 0.01) with the different viruses and the presence of virus in tissue culture supernatants was quantified at different hours post-infection (h.p.i.). No statistically significant differences were observed between the replication kinetics of the rBTV-1, rBTV-4, and rBTV-8 viruses compared to their respective WT viruses (Fig. 1b). The expression of reporter genes did not critically compromised virus replication in Vero cells. Nonetheless, incorporation of Venus and NLuc seemed to be related with lower replication levels of rBTV-4/Venus and rBTV-4/NLuc compared to those displayed by WT BTV-4 and rBTV-4. A similar effect in replication for rBTV-8/Venus and rBTV-8/NLuc was also observed (Fig. 1b). In addition, over the same time course of 24–72  h.p.i., we evaluated NLuc activity in collected supernatants (Fig. 1c). Notably, NLuc activity was detected as early as 24 h.p.i. (first time point measured) and showed a time-dependent increase of activity, peaking at 72 h.p.i., most likely because the cytopathic effect (CPE) caused by viral infection resulted in the release of NLuc protein in the culture supernatants (Fig. 1c).

Although the plaque size is very heterogeneous for all viruses, when we evaluated the plaque phenotype in Vero cells, we observed that plaques generated by rBTV-8/Venus (0.91 ± 0.2 mm^2^) and rBTV-8/NLuc (0.39 ± 0.15 mm^2^) were significantly smaller than plaques formed after infection with WT BTV-8 (1.81 ± 0.15 mm^2^). However, we observed similar plaque sizes for the other analyzed viruses (BTV-1 WT: 2.29 ± 0.15 mm^2^; rBTV-1: 2.60 ± 0.2 mm^2^; rBTV-1/Venus 2.36 ± 0.2 mm^2^; rBTV-1/NL 2.20 ± 0.25 mm^2^; BTV-4 WT: 1.62 ± 0.35 mm^2^; rBTV-4: 1.50 ± 0.22 mm^2^; rBTV-4/Venus 1.32 ± 0.16 mm^2^; rBTV-4/NL 1.11 ± 0.15 mm^2^) (Fig. 1d).

In addition, the expression of reporter genes was confirmed by Western blotting (Fig. 1e). Cell extracts from either mock or infected (MOI 0.01) Vero cells were tested at 48 h.p.i using antibodies specific for viral NS2, green fluorescent (GFP; for Venus expression), and NLuc proteins. Western blot analysis showed specific bands with the expected molecular sizes for NLuc or Venus proteins in cell extracts from reporter-expressing rBTV-infected cells (Fig. 1e). In addition, we also observed specific bands for NS1-2A-NLuc and NS1-2A-Venus fusion proteins, indicating that the efficiency of 2A autoprotease peptide sequence used is not 100%, as previously described (67, 68). Overall, these data indicate that the expression of Venus or NLuc reporter genes did not significantly disrupt the viral fitness *in vitro*.

**Fig 2:**
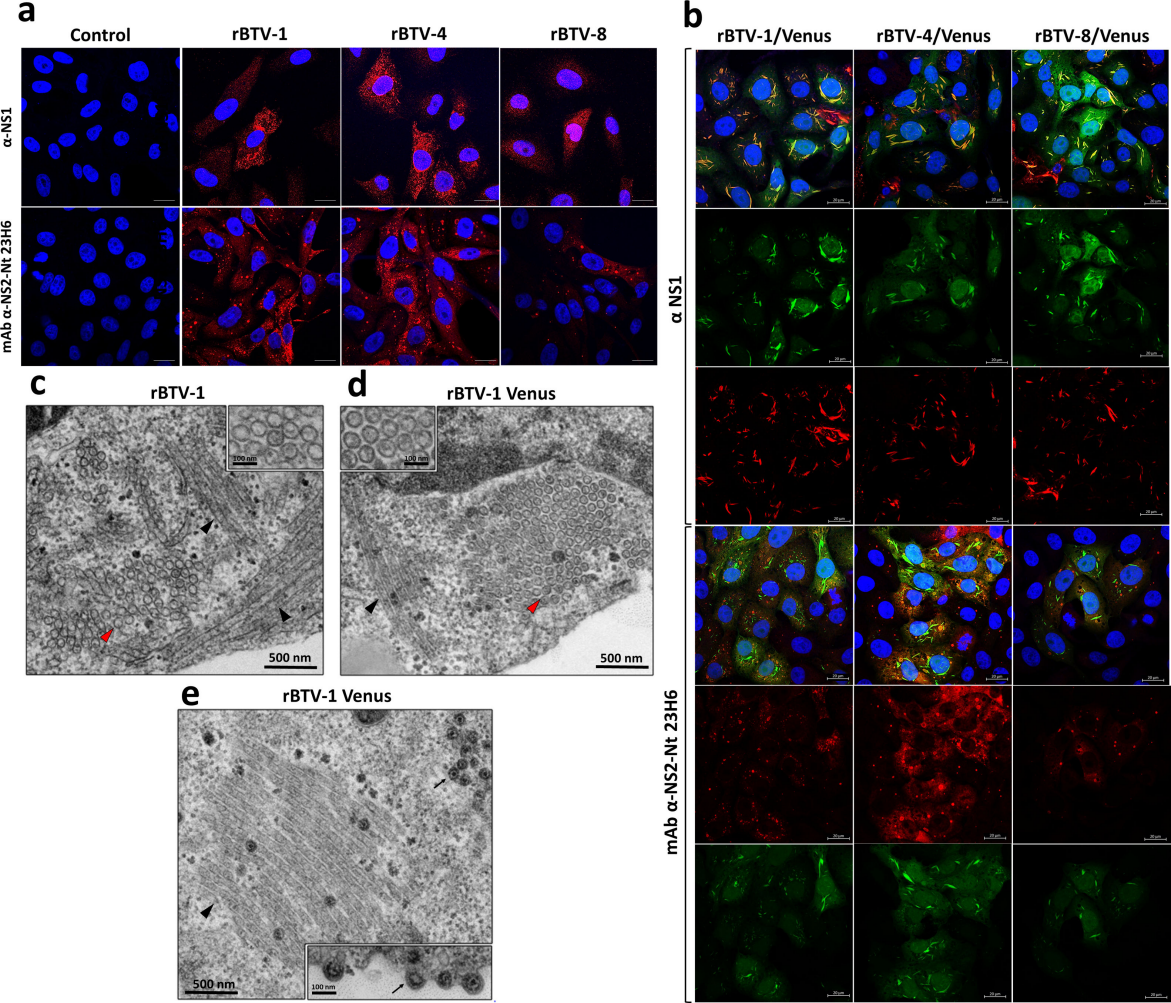
Formation of microtubules in cells infected with reporter-expressing rBTV. (**a**) Confocal microscopy. Indirect immunofluorescence of Vero cells infected (MOI of 0.1) with (**a**) rBTV-1, rBTV-4, rBTV-8, or non-infected and (**b**) rBTV-1/Venus, rBTV-4/Venus, rBTV-8/Venus, or non-infected. NS1 was detected using a mouse polyclonal hyperimmune serum against NS1 (red). NS2-Nt was detected using a mouse MAb (red). Venus expression was directly visualized in green. Nuclei were stained with DAPI (blue). Bars, 20 µm. (**c–e**) Electron microscopy. Semiconfluent Vero cell monolayers were infected with rBTV-1 (**b**) or rBTV-1/Venus (MOI of 1) (**c and d**). After 24 h, cells were fixed and at 24 h and processed for transmission electron microscopy. Arrow heads indicate longitudinal (black) and transversal cross (red) sections of NS1 microtubules, which are shown at higher magnification in the upper insets. Black and blue arrows in **d** indicate BTV particles. The lower inset in panel d shows BTV particles budding at the plasma membrane virions. Bars, 500 nm (**c–e**), 100 nm (insets in c–**e**).

The expression of the reporter gene Venus was also analyzed in cell culture by confocal microscopy (Fig. 2a). Vero cells were mock-infected or infected (MOI 0.1) with rBTV-1, rBTV-1/Venus, rBTV-4, rBTV-4/Venus, rBTV-8, or rBTV-8/Venus, and the expression of Venus in the infected cells was examined at 24 h.p.i. Antibodies recognizing viral NS1 or NS2 proteins were also used as control of infection. As expected, Venus expression was observed in all infected cells, as deduced from double labeling with either anti-NS1 or anti-NS2 antibodies. Venus fluorescence was detected in the cytoplasm of the cells infected with rBTV/Venus but also a clear co-localization between Venus and NS1 protein was detected which is consistent with an incomplete processing of the fusion protein formed by NS1 and the reporter Venus protein (see also Fig. 1e). Interestingly, the immunofluorescence of NS1 in the cells infected with Venus-expressing rBTVs displayed a filamentous pattern that was different from the typical NS1 punctate signal observed in rBTV-infected cells.

It is well known that NS1 rapidly multimerizes to form cytoplasmic tubular assemblies, which is considered a hallmark of cellular infections by orbiviruses (56, 57). To determine if the modified NS1 sequence of the reporter-expressing rBTV affects the formation of NS1 tubules, Vero cells infected for 24 h with rBTV-1 or rBTV-1/Venus (MOI 1) were analyzed by transmission electron microscopy. As shown in Fig. 2b and c, large bundles of cytoplasmic NS1 tubules were detected in both infections, showing no significant differences in their diameters (rBTV-1: 54.0 ± 3.4 nm; rBTV-1/Venus: 53.6 ± 2.9 nm, *n*: 65 per condition, *P* > 0.50). As expected, rBTV-1/Venus-infected cells produced large amounts of intracellular and extracellular virus particles (Fig. 2d). This indicate that the full-length NS1-2A-Venus (and also NS1-2A) protein does not appear to significantly alter NS1 tubule formation or virus assembly.

### *In vitro* stability of reporter-expressing rBTV

**Fig 3:**
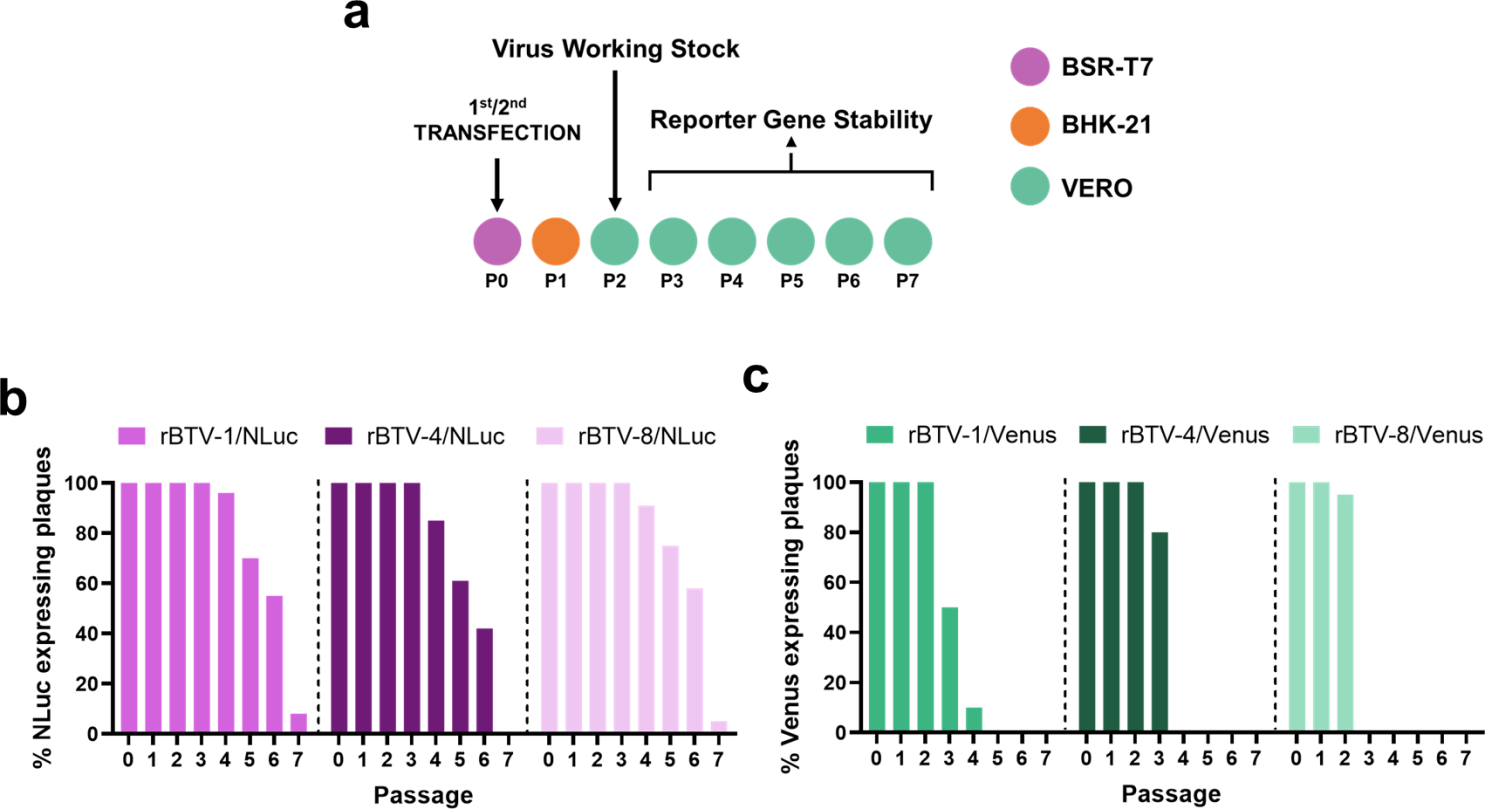
Genetic stability of reporter-expressing BTV *in vitro*. (**a**) After generation of virus working stocks (**P2**), recombinant viruses were passaged up to five times in Vero cells (P3–P7). Stability is expressed as the percentage of plaques (*n* = 30) that expressed NLuc (**b**) or Venus (**c**) reporter genes in each passage.

Genetic and phenotypic stability of reporter-expressing viruses is a significant concern that can limit their applications (44, 45). For that, we evaluated the *in vitro* genetic stability of the reporter-expressing rBTV (Fig. 3). To that end, rBTV-1/NLuc, rBTV-1/Venus, rBTV-4/NLuc, rBTV-4/Venus, rBTV-8/NLuc, and rBTV-8/Venus from the generated working stocks (P2) were passaged five consecutive times in Vero cells, and the percentage of reporter-expressing viruses was determined by plaque assay (Fig. 3a). In the case of Venus-expressing viruses, plaques were observed directly under a fluorescence microscope. For the expression of NLuc, plaques were amplified in 96-well plates, and then, NLuc activity in the tissue culture supernatant was evaluated at 48 h.p.i. NLuc-expressing rBTV (Fig. 3b) showed higher stability than Venus-expressing viruses (Fig. 3c). Although reporter gene expression is eventually lost by serial passage in cell culture, viruses that express Venus or NLuc are 100% stable until passage two (virus working stock), and gradual loss of expression of the Venus reporter gene and the NLuc reporter gene is observed from passage 3 and from passage 4, respectively.

### Pathogenicity and replication of NLuc-expressing rBTVs in IFNAR(−/−) mice

**Fig 4:**
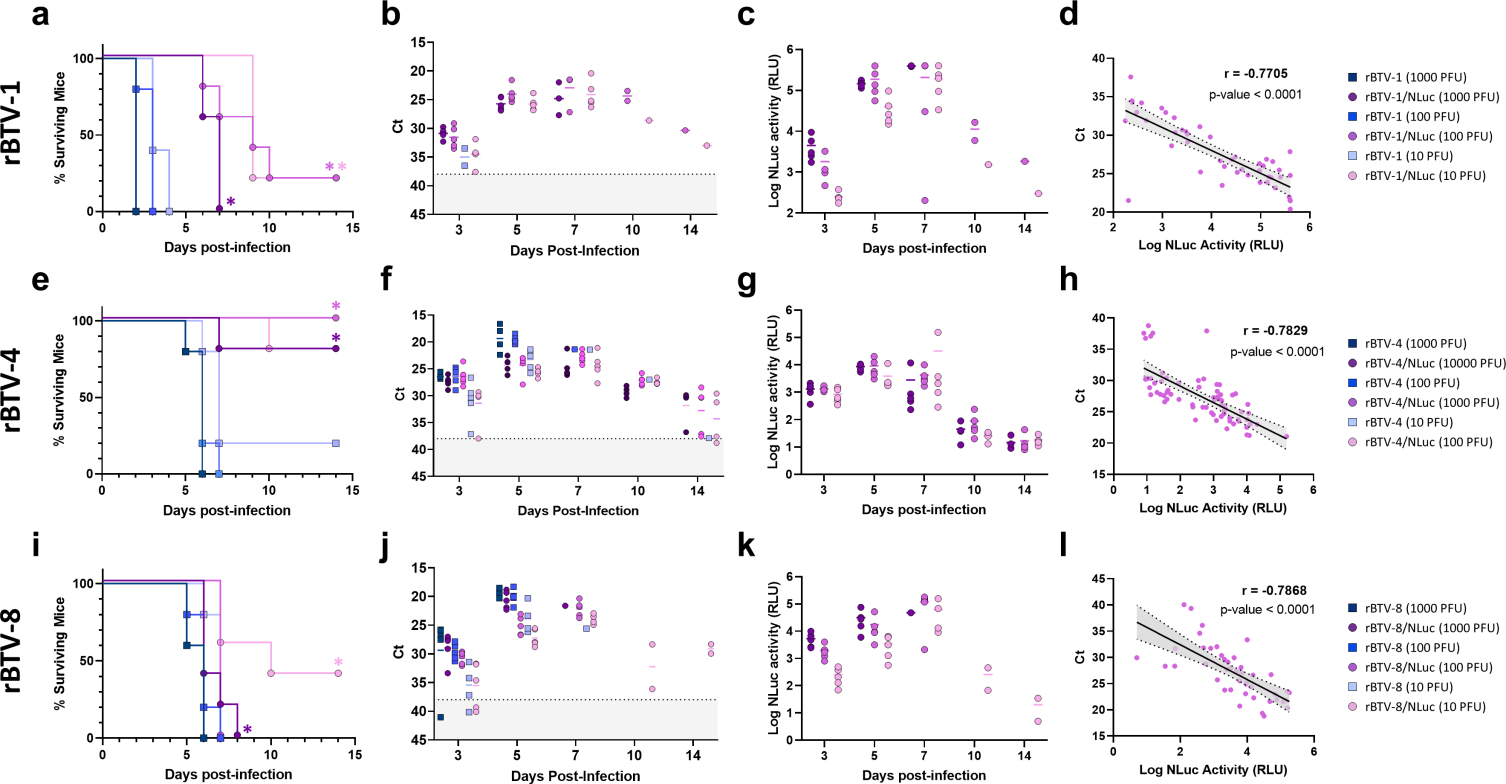
Pathogenesis and replication of NLuc-expressing rBTVs in Imice. Groups of IFNAR(−/−) mice (*n* = 5) were inoculated with a dose of 10, 100, or 1,000 PFU of rBTV-1, rBTV-1/NLuc, rBTV-4, rBTV-8, or rBTV-8/NLuc. For rBTV-4/NLuc, doses of 100, 1,000, or 10,000 PFU were used in the experiment. (**a, e, i**) Survival rates after infection. Statistical differences between curves of NLuc-expressing rBTV and their corresponding wild-type recombinant viruses were calculated by Log-rank test (*P* < 0.05). (**b, f, j**) RNAemia analyzed by RT-qPCR after viral inoculation. Expression of mRNA of segment 5 was quantified at 3, 5, 7, 10, and 14 d.p.i. Results were expressed as Ct (left *y* axis). The real-time RT-qPCR specific for BTV segment 5 was performed as described by Toussaint *et al*. (83). Cut-off Ct ≥ 38 (dotted gray line). Points represent individual Ct value for each mouse, and lines of the corresponding color represent the mean Ct value of each group. No statistical differences were found between groups as calculated by multiple *t* test analysis using the Holm-Šídák method. (**c, g, k**) Luminescence activity in sera from rBTV inoculated mice. Points represent individual signal for each mouse and lines of the corresponding color represent the mean signal value of each group. No statistical differences were found between groups as calculated by multiple *t* test analysis using the Holm-Šídák method. (**d, h, l**) Correlation between RNAemia levels and luminescence signals after viral inoculation as calculated by Spearman’s rank order correlation.

Fluorescent reporters are useful for tracking viral replication at the cellular level. However, fluorescent protein expression has several limitations for *in vivo* studies, and luciferase reporters are the preferred option to study viruses using whole animals. For that reason, we next evaluated if NLuc-expressing rBTV is fully infectious, pathogenic, and able to replicate in a mouse model of BTV infection (Fig. 4). Groups of adult male IFNAR(−/−) mice (*n* = 5) were inoculated intraperitoneally with different viral doses of rBTV-1, rBTV-1/NLuc rBTV-4, rBTV-4/NLuc, rBTV-8, or rBTV-8/NLuc, and mortality was monitored for 14  days post-infection (d.p.i.) (Fig. 4a, e and i). In addition, RNAemia was evaluated at 3, 5, 7, 10, and 14 d.p.i. by PCR-testing (Fig. 4b, f and j). Notably, mice inoculated with NLuc-expressing rBTV showed similar clinical signs as those inoculated with rBTV, including lethargy, ruffled hair, and conjunctivitis, previously described for BTV infection (69). For rBTV-1, all mice from groups inoculated with rBTV-1 died during the first days after infection (Fig. 4a). Similarly, mice that received the highest dose (1,000 PFU) of rBTV-1/NLuc succumbed to infection by day 7 post-infection. Nonetheless, lower doses of rBTV-1/NLuc (10 or 100 PFU) also showed a high mortality rate of 80% (one mouse survived in both groups). Regarding RNAemia levels by Ct values (Fig. 4b), a peak was observed for all three rBTV-1/NLuc mice groups at 7 d.p.i., with the surviving mice nearly PCR-negative at the end of the experiment. Administration of 100 or 1,000 PFU of rBTV-4 produced a 100% mortality rate by 6 or 7 d.p.i., while one mouse survived from the group inoculated with 10 PFU of rBTV-4 (Fig. 4e). In view of a hypothesized *in vivo* attenuation based on the replication kinetics and diminished plaque sizes of rBTV-4/NLuc, a 10 times higher dose was used. Indeed, inoculation with 100; 1,000; or 10,000 PFU of rBTV-4/NLuc did not cause fatal disease in all animals, and only one mouse from the rBTV-4/NLuc 100 PFU group and another animal from the rBTV-4/NLuc 10,000 PFU group succumbed to infection (Fig. 4e). However, rBTV-4/NLuc inoculated mice displayed marked clinical signs of viral disease, and the observed RNAemia profile was very similar to those of the rBTV-4 inoculated mice groups. RNAemia peaked between days 5 and 7 post-infection and was cleared at 14 d.p.i. (Fig. 4f), as observed for the unique surviving mice inoculated with rBTV-4. Inoculation with doses of 10, 100, or 1,000 PFU of rBTV-8 resulted in a mortality rate of 100%, with all mice dying between day 6 and 7 post-infection. In contrast, inoculation of 10 PFU of rBTV-8/NLuc led to a mortality rate of 60% at the end of the experiment. However, doses of 100 and 1,000 PFU of rBTV-8/NLuc killed all mice at the same days as the equivalent doses of rBTV-8 did (Fig. 4i). Viral RNA levels were dose-dependent and very similar between groups, with peak levels at 5 d.p.i. (Fig. 4j). Overall, these data confirm that NLuc-expressing rBTVs can lead to a lethal infection in mice although they show some attenuation with respect to rBTVs without NLuc.

Since BTV causes viraemia in infected animals and the virus can be isolated from blood, we speculated that the presence of NLuc could be detected in blood samples from infected animals. To prove this hypothesis, NLuc activity was measured from plasma extracted from the blood samples. Notably, NLuc activity was detected in the plasma of all the animals infected with NLuc-expressing rBTVs (rBTV-1/NLuc, rBTV-4/NLuc, and rBTV-8/NLuc) (Fig. 4c, g, and k). More importantly, NLuc activity in plasma samples was dose-dependent, showing a peak signal coinciding with RNAemia peak levels in all cases. Furthermore, very low NLuc activity was measured in plasma of those animals that nearly recovered from the infection at the end of the experiment. Moreover, NLuc activity inversely correlated with Ct values, thus positively correlated with RNAemia levels, for rBTV-1/NLuc (Pearson *r* = −0.7705; *P*-value < 0.0001), rBTV-4/NLuc (Pearson *r* = −0.7829; *P*-value < 0.0001), and rBTV-8/NLuc (Pearson *r* = −0.7868; *P*-value < 0.0001) (Fig. 4d, h, and l). This demonstrates that NLuc activity is an easy and rapid measurement as a potential biomarker of virus replication *in vivo.* Altogether, these findings indicate that NLuc-expressing rBTV can be used to evaluate viral infection and replication *in vivo*.

### NLuc-expressing rBTV for preclinical evaluation of vaccines

**Fig 5:**
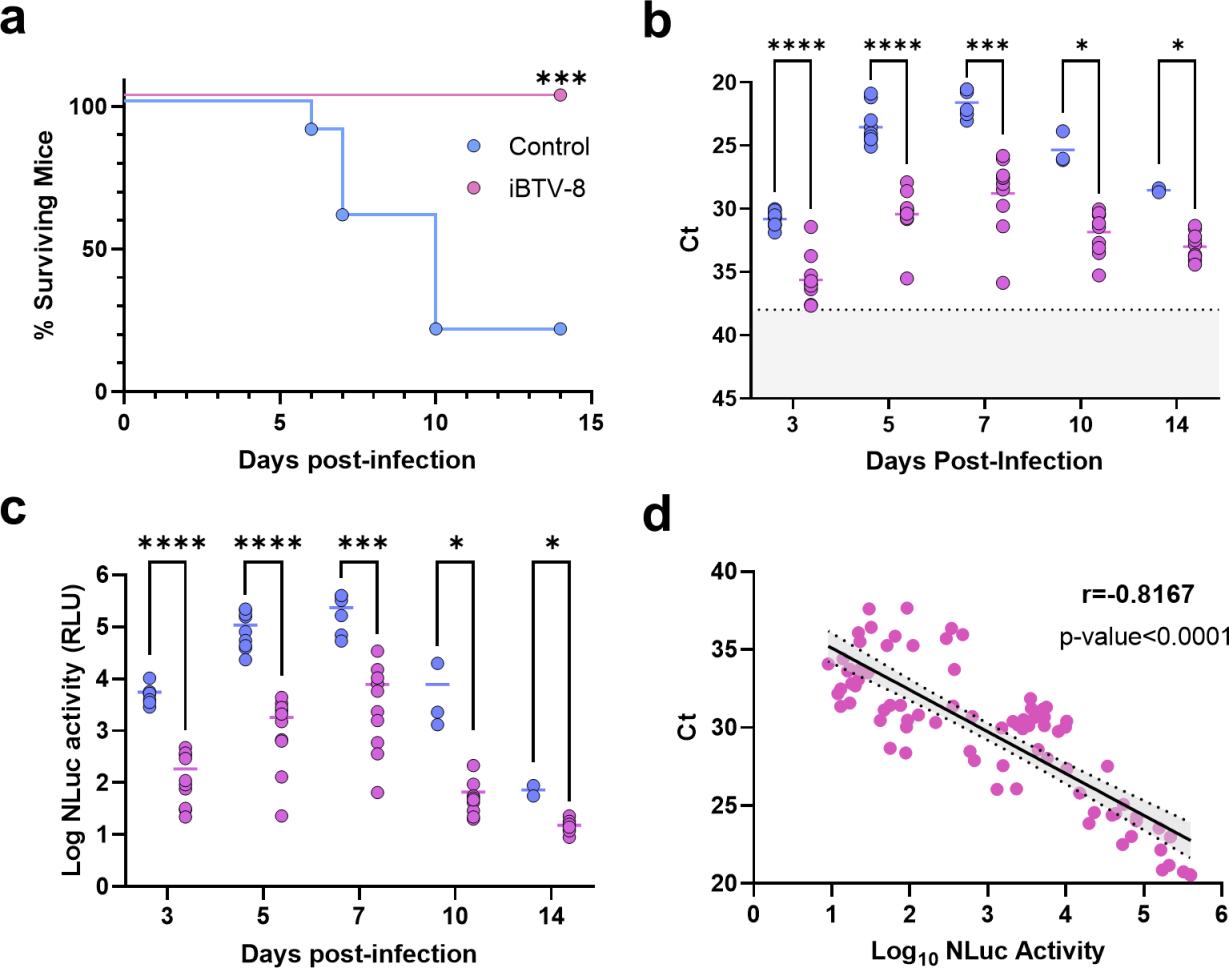
Evaluation of vaccine efficacy by measuring NLuc expression in plasma samples. Groups of IFNAR(−/−) mice (*n* = 10) were inoculated with two doses of a commercial inactivated vaccine (iBTV-8) against BTV-8 and challenged with 1,000 PFU of rBTV-8/NLuc. A group of mice was non-immunized (control). (**a**) Survival rates after infection. Statistical differences between curves were calculated by Log-rank test (***P < 0.0002). (**b**) RNAemia analyzed by RT-qPCR after viral inoculation. Expression of mRNA of segment 5 was quantified at 3, 5, 7, 10, and 14 d.p.i. Results were expressed as Ct (left *y* axis). The real-time RT-qPCR specific for BTV segment 5 was performed as described by Toussaint *et al*. (83). Cut-off Ct ≥ 38 (dotted gray line). Points represent individual Ct value for each mouse, and lines of the corresponding color represent the mean Ct value of each group. Differences between groups were calculated by multiple *t* test analysis using the Holm-Šídák method. (**c**) Luminescence activity in sera from inoculated mice. Points represent individual signal for each mouse, and lines of the corresponding color represent the mean signal value of each group. Differences between groups were calculated by multiple *t* test analysis using the Holm-Šídák method. *P < 0.05; ***P < 0.0002; ****P < 0.0001. (**d**) Correlation between RNAemia levels and luminescence signals after viral inoculation as calculated by Spearman’s rank order correlation.

Preclinical efficacy studies for novel vaccine candidates can be time-consuming and expensive, and they involve the use of traditional methods to quantify virus replication such as plaque assays, qPCR, TCID_50_ (Median Tissue Culture Infectious Dose), etc. (61, 62, 70, 71). As a proof of concept, bioluminescent reporter virus rBTV-8/NLuc was used to assess the vaccine efficacy. IFNAR(−/−) mice (*n* = 10) were immunized with a commercial inactivated vaccine against BTV-8 (72) following a homologous prime-boost regime administered 4 weeks apart. An additional group of IFNAR(−/−) mice (*n* = 10) was left untreated. Four weeks after the boost immunization, both groups of animals were challenged with a lethal dose of rBTV-8/NLuc (1000 PFU) and mice were daily monitored for signs of disease and survival (Fig. 5a). Moreover, at 3, 5, 7, 10, and 14 d.p.i., viral replication (Fig. 5b) and NLuc activity (Fig. 5c) were determined in blood and plasma samples, respectively. As expected, immunization with the commercial vaccine was fully protective, preventing illness or death in vaccinated mice, while most of mock-vaccinated mice succumbed to the challenge and only two mice survived (Fig. 5a). Consistent with these observations, viral replication (Fig. 5b) and NLuc measures (Fig. 5c) were significantly higher in mock-vaccinated mice at all the time points evaluated. Importantly, the bioluminescent signal kinetics in plasma samples were consistent and correlated with virus titer kinetics (Pearson *r* = −0.8167; *P*-value < 0.0001) (Fig. 5d). This demonstrates that, at least in mice, NLuc-expressing rBTVs are a suitable biotechnological instrument for the rapid and easy preclinical assessment of novel vaccines against BTV.

### Real-time dynamics of rBTV-1/NLuc in a mouse model of infection

**Fig 6:**
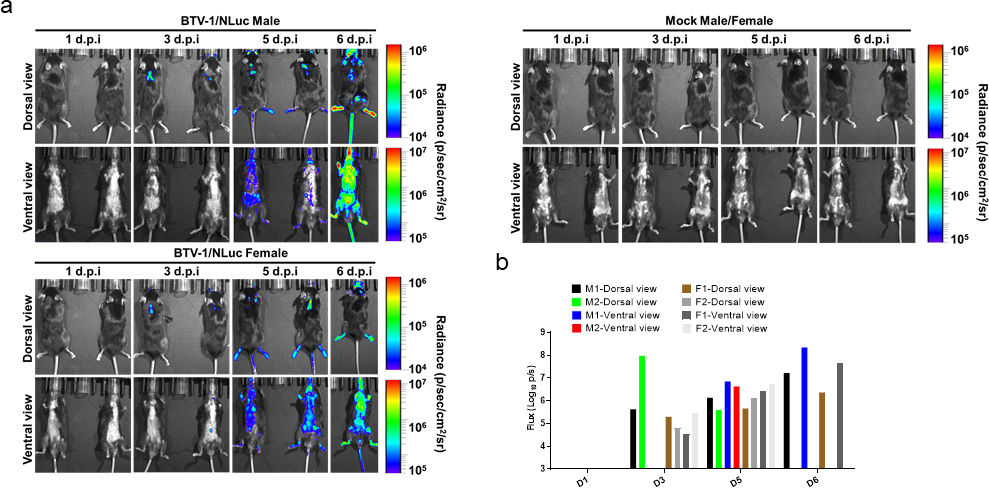
*In vivo* kinetics of rBTV-1/NLuc infection by real-time monitoring of NLuc expression. IFNAR(−/−) mice were mock-infected (one male and one female) or inoculated with 10^3^ PFU of rBTV-1/NLuc (two males and two females). NLuc activity in the whole mouse was evaluated on the indicated days p.i. (1, 3, 5, and 6). (**a**) Images of the mice for each time point show the radiance (number of photons per second per square centimeter per steradian [p/sec/cm2/sr]). (**b**) Bioluminescence values were quantified, and the total flux [in log_10_ (number of photons per second) (p/s)] from ROIs (Region of interest) is presented.

For *in vivo* studies using living animals, luciferase reporter proteins are the preferred option because fluorescent proteins are normally disturbed by the nonspecific background fluorescence associated with live tissues (44, 47, 48, 73–75). Therefore, to evaluate the dynamics of BTV *in vivo*, male and female IFNAR(−/−) mice were mock-infected or infected with 1,000 PFU of rBTV-1/NLuc. Animals were monitored daily for signs of disease and survival and bioluminescent signal was assessed at 1, 3, 5, and 6 d.p.i. (Fig. 6a). Only two mice (one male and one female) survived until day 6 post-infection. We were able to delineate the dynamics of rBTV-1/NLuc infection by bioluminescence imaging, and we detected a luminescent signal as early as 3 d.p.i. (Fig. 6a). *In vivo* imaging of NLuc expression was initially detected in the skin at the site of infection and the lymph nodes and later, after systemic spread occurred, in the spleen, lung, thymus, and ovaries, target tissues for BTV replication in IFNAR(−/−) mice and ruminants (Fig. 6a and b). Bioluminescence intensity reached maximum values at 5–6 d.p.i. in male and female mice. Thus, rBTV-1/NLuc luciferase expression dynamics correlated well with viral replication, which peaked at 5 d.p.i. (Fig. 4). Furthermore, NLuc expression increased over time until mice succumbed to infection.

**Fig 7:**
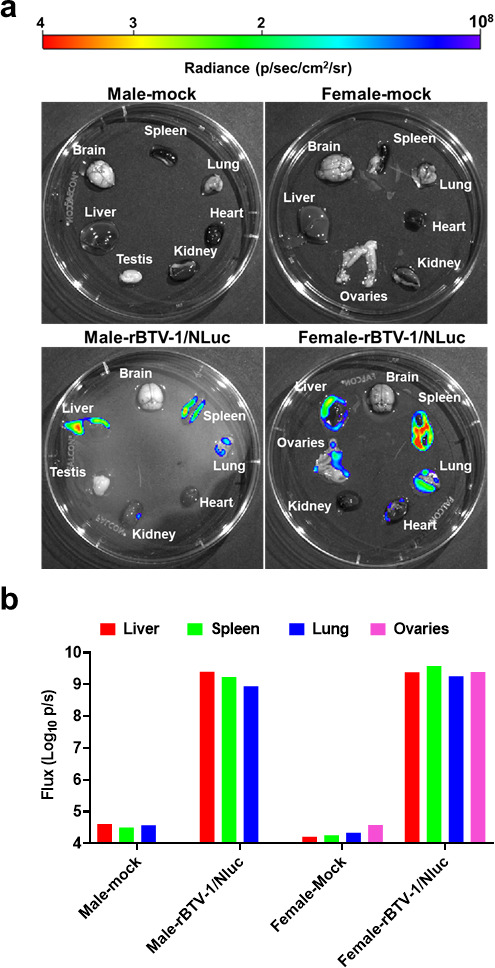
rBTV-1/NLuc propagation in animal organs. Mice from day 6 post-infection (Fig. 6) mock infected (one male and one female) or infected (one male and one female) were sacrificed, and the brain, spleen, lungs, heart, kidney, liver, and testis/ovaries were removed in one piece, washed, and examined by IVIS to assess for NLuc activity. Images of whole organs (**a**) and NLuc quantification (**b**) are indicated. Radiance (number of photons per second per square centimeter per steradian [p/sec/cm^2^/sr]). The average total flux [in log_10_ (number of photons per second) (p/s)] is shown.

To evaluate the distribution of the virus, infected animals (one male and one female) were euthanized at day 6 post-infection together with mock-infected animals as controls. Following sacrifice, the brain, spleen, lung, heart, kidney, liver, and testis/ovaries were immediately removed in one piece, washed, and examined by *in vivo* imaging systems (IVIS) to assess bioluminescence. Similar to our previous results based on viral replication (76), the highest NLuc expression was detected in the lung, spleen, ovaries, and liver also, whereas marginal background luminescence was noted in mock-infected animals (Fig. 7).

## DISCUSSION

Reporter-expressing, replication-competent viruses have represented an excellent tool for basic and/or translational studies (44, 45, 77–79). To date, one modified replication-competent rBTV-1 that expresses the fluorescent mCherry fused to the NS3 protein and a rBTV-8 that expresses eGFP fused to the NS1 protein have been described (80, 81). Therefore, designing novel strategies for engineering improved reporter-expressing rBTV would undoubtedly enhance knowledge on BTV (and related orbiviruses) replication and pathogenesis. Fluorescent and bioluminescent proteins are the most extensively used reporter proteins. However, due to their different properties, the use of these reporter genes is limited to specific experimental approaches, which need to be carefully considered before performing the experiment. To overcome this limitation, here, we have designed and generated new reporter-expressing rBTV encoding Venus fluorescent or NLuc luciferase proteins. In addition, multiple rBTV representing currently main circulating serotypes in Europe were generated. Importantly, our work provides valuable evidences regarding the plasticity of the BTV genome to accommodate and express large ORFs from the viral segment 5.

The expression of Venus and NLuc was confirmed directly in culture cells using a microplate reader (NLuc) or a fluorescence microscope (Venus). Venus and NLuc expression were further confirmed by Western blotting, showing a specific band of the reporter protein and the NS1-2A-reporter fusion polyprotein. Although the peptide 2A is highly active in mediating cleavage, polyproteins containing self-cleaving 2A produce three translational products, the processed proteins, and the non-cleaved polyprotein (82). A characteristic of orbivirus infections is the accumulation of cytoplasmic tubular structures composed of NS1 (57, 58). When we compared the presence of tubules in Vero cells infected with rBTV-1 or rBTV-1/Venus, no differences were observed regarding the presence or diameter of tubules in the cytoplasm. Apparently, the full length NS1-2A-Venus protein does not alter NS1 tubule formation.

In Vero cells, reporter-expressing rBTV replicated slightly slower than the WT and rBTV, resulting in slightly lower viral titers too. Previously, Shaw et al. showed that BTV-1/NS3mCherry displayed a lower replication rate in BSR, BFAE, and KC cells compared to WT BTV-1, specially in BFAE and KC cells (80). In this rBTV-1, the reporter gene mCherry was fused to the segment 10, and their data suggest that the function of NS3 on BTV egress is partially compromised in the NS3mCherry fusion protein. In addition, passaging of BTV-1/NS3mCherry in BSR cells leads to the selection of deletion mutants that lack the intact mCherry reading frame. In the case of the recombinant BTV-8 that expresses eGFP fused to the NS1, the virus is stable up to two passages in cell culture, but its growth kinetics has not been compared with the WT BTV-8 nor its virulence *in vivo* has been tested (81). To avoid potential negative effects of a direct fusion to protein NS1, the reporter genes were inserted in the viral segment 5 downstream of a PTV-1 (porcine teschovirus-1) 2A cleavage site (44, 82–84). This system results in the individual expression of the NS1 and the reporter gene from the same ORF and viral segment by a translational effect that is known as “stop-go” or “stop-carry” (85). The disadvantage of this strategy is that it cannot provide information about the localization of the viral protein to which it was fused. Although the strategy that we used to generate rBTV/Venus and rBTV/NLuc implies independent translation of BTV segment 5 and the reporter genes, the presence of full-length NS1-2A-reporter gene could affect NS1 functions, thus justifying the slight reduction in virus growth *in vitro* and the attenuation *in vivo*. This is also supported by the eventual loss of the reporter gene, which indicates the positive selection of mutants lacking the reporter gene. In any case, the use of PTV-1 2A self-cleavage site to separate the viral and reporter proteins has been shown to be more successful than the direct fusion of the reporter genes for Lassa Virus (LASV) (86), lymphocytic choriomeningitis virus (LCMV) (87, 88), or Lujo hemorrhagic fever virus (LUHFV) (89). It has also been described for arenaviruses that both the size and location of the reporter genes have an impact on growth and viability of the recombinant viruses (90). The smaller size of NLuc (19.1 kDa) compared to Venus (26 kDa), or their physicochemical properties could be one of the reasons why the rBTV/NLuc were observed more stable than rBTV/Venus in cell culture.

One of the main limitations of recombinant viruses expressing foreign genes is their potential attenuation *in vivo* (44, 84, 91). Therefore, to assess whether the expression of NLuc affects BTV replication and virulence *in vivo*, we infected BTV-susceptible IFNAR(−/−) mice. Despite encoding a reporter gene and displaying a slight reduction in virus growth *in vitro*, NLuc-expressing rBTVs retained their virulence although they were slightly attenuated compared to rBTV, especially rBTV-4/NLuc. In any case, although in infection with rBTV-4/NLuc the clinical signs are attenuated, the virus replicates in the animal and RNAemia can be detected. Most likely, in the case of rBTV-4/Nluc, a higher viral dose should be used for the challenge studies to improve its use in vaccine testing. NLuc activity was measured in plasma samples from infected IFNAR(−/−) mice, and more importantly, the bioluminescent signal strongly correlated with RNAemia as measured by PCR testing. We here demonstrated the feasibility of using this reporter gene as a valid surrogate of viral infectivity and replication. This feature allowed us to use rBTV-8/NLuc in order to study the efficacy of a marketed vaccine against BTV-8. Measurement of viral replication by qPCR or other laborious conventional assays, like virus isolation, usually take days. The possibility of quantifying bioluminescence directly from blood samples avoids time-consuming sample manipulation steps. This allows the accurate and rapid determination for the presence of virus, which could pave the way to rapidly evaluate novel vaccine candidates. A next step could be to measure NLuc activity upon infection of larger animals such as sheep, one of the natural hosts of BTV.

Besides, we studied the dynamic of BTV infection in mice using IVIS technology. NLuc quantification in living animals allowed us to longitudinally analyze BTV infection dynamics in the same animal during the natural progression of viral infection. Moreover, postmortem studies showed NLuc activity in multiple organs surgically collected from infected animals. This is in agreement with observed virus spread to target BTV organs as spleen, thymus, or lung during infection (76). NLuc is present in the plasma of infected animals and BTV replicates in white blood cells (69, 92). This might limit studies on organ specificity and pathogenesis and could be the cause of the observed luminescent signal distributed throughout the animal at late post-infection times. In any case, these mice studies have demonstrated that NLuc-expressing rBTV are also a powerful biotechnological tool to track and visualize infectivity *in vivo* and at postmortem examination.

Overall, we have demonstrated the feasibility and adaptability of our system to generate reporter-expressing rBTV to perform different *in vitro* and *in vivo* studies. Many virus species within the *Orbivirus* genus cause severe disease in mammals. However, three Culicoides-transmitted arboviruses have gathered more attention due to their significantly high economic impact, namely, BTV, African horse sickness virus (AHSV), and epizootic hemorrhagic disease virus (EHDV). Several serotypes of BTV and AHSV have been reported in Europe, and outbreaks of EHDV-8 were reported for the first time in Europe in 2022 (93–96). The studies described in this work will be very useful to evaluate pathogenicity of these highly relevant orbiviruses and will contribute to the development of novel therapeutic alternatives, such as new vaccines and antivirals.

## MATERIALS AND METHODS

### Cells lines and viruses

Green monkey kidney cells (Vero) (Vero; ATCC catalog no. CCL-81) and Baby hamster kidney cells (BHK-21; ATCC catalog no. CCL-10) were grown in Dulbecco’s modified Eagle’s medium (DMEM) supplemented with 5% heat-inactivated fetal bovine serum (FBS), and 1% PSG (penicillin, 100 units/mL; streptomycin 100 µg/mL; l-glutamine, 2 mM) at 37°C in air enriched with 5% CO_2_.

BSR-T7 cells (97) stably expressing bacteriophage T7 RNA polymerase were maintained in the same manner as above except for the addition of 1 mg/mL of Geneticin every second passage.

Wild-type BTV serotype 1 (ALG2006/01) (BTV-1), BTV serotype 4 Morocco strain (MOR2009/09) (BTV-4M), and BTV serotype 8 (BEL/2006) (BTV-8) were used in this study. BTV-4M Morocco strain (MOR2009/09) is a reassortant between BTV-1 (segments 1, 4, 5, 7, 9, 10) and BTV-4 (segments 2, 3, 6, 8) isolated from sheep blood in KC insect cells and generously provided by The Pirbright Institute, UK (69, 98). Virus stocks were propagated in Vero cells and titrated by plaque assay. Briefly, 10-fold dilutions of virus stocks were inoculated into 12-well plates containing semi-confluent monolayers of Vero cells. Following incubation for 1 h, an agar overlay [DMEM, 10%-FBS, 0.4%-Noble Agar (Becton Dickinson)] was added and plates were incubated for 5 days at 37°C in 5% CO2. Plaques were fixed with 10% formaldehyde and visualized with 2% crystal violet-PBS. The viral titer of the stock sample was determined by taking the average number of plaques for a dilution and the inverse of the total dilution factor.

### Plasmids

Expression plasmids containing ORFs for VP1, VP3, VP4, VP6, NS1, and NS2 of BTV6/net08 has been previously described (22). These plasmids were used to rescue all the recombinant viruses used in this study.

All pUC57 RG vectors were designed to contain the viral cDNAs (Table 1) flanked upstream by a T7 RNA polymerase promoter sequence and downstream by the hepatitis delta virus ribozyme (HDVR) and T7 RNA polymerase terminator sequences. In addition, an *Xho*I restriction enzyme site was incorporated into the cDNA of segment 8 by the introduction of two silent point mutations (T635C and G637C). In order to generate replication-competent reporter-expressing BTVs, we engineered a recombinant segment 5 containing the viral NS1 ORF, without the stop codon, followed by a linker (GSG), the porcine teschovirus-1 (PTV-1) 2A autoproteolytic cleavage site (ATNFSLLKQAGDVEENPGP), and a multicloning site (*Age*I*/Bgl*II*/Nhe*I) for the insertion of foreign sequences. Importantly, 5′- and 3′-untranslated regions (UTRs) were not modified. This master plasmid (named pUC57-S5/2A) can be used to introduce different foreign genes. All sequences were synthetized *de novo* (Biomatik or Genscript) and assembled in pUC57 RG vectors by the same companies. The Venus or NLuc reporter genes were amplified by PCR using specific primers containing *AgeI* (Venus/*Age*I/F: AATT*ACCGGT*atggtgagcaagggcgaggagctg and NLuc/*AgeI*/F: AATT*ACCGGT* atggtcttcacactcgaagatttc) or *NheI* (Venus/*NheI*/R: AATT*GCTAGC*ttacttgtacagctcgtccatgcc and NLuc/*NheI*/R: AATT*GCTAGC* ttacgccagaatgcgttcgcacag) sites. Then, PCR products and pUC57-S5/2A plasmid were digested, and the reporter genes were cloned using standard molecular biology techniques. Plasmid constructs were confirmed by sequencing (Macrogen).

### Rescue of recombinant viruses

To rescue BTV, monolayers of BsrT7 cells (T25 flasks, 50% of confluence) were first co-transfected with 1 µg of each of the expression plasmids (VP1, VP3, VP4, VP6, NS1, and NS2 ) (22). Then, at 24 h post-transfection (h.p.t.), cells were further co-transfected with 1 µg of each of the RG plasmids encoding the BTV cDNAs. Both transfection steps were performed using Lipofectamine 3000 (Invitrogen) in Opti-Mem I Reduced Serum Medium (Invitrogen) according to the manufacturer’s instructions. Four hours after the second transfection, the transfection mix was replaced with 6 mL DMEM supplemented with 5% FBS. Then, 48–72 h later, cell culture supernatants were collected, clarified, and used to infect fresh monolayers of BHK-21 cells (T25 flasks, 50% of confluence). Once the cytopathic effect (CPE) was observed (48–72 h), the medium was harvested and clarified. Virus recovery was confirmed by infection of BHK-21 cell monolayers with the cell culture supernatants and immunofluorescence assay at 24 h post infection as previously described (61, 62, 99). All viral stocks were prepared in Vero cells, and virus titer was determined by plaque assay as previously described (98). A list of the recombinant viruses rescued and their gene constellations is included in Table 2. Commercial diagnostic tool VetMAX European BTV Typing Kit (Thermo Fisher Scientific) was used for BTV serotyping, following manufacturer’s indications. In addition, the segment 8 was partially sequenced because a *Xho*I restriction enzyme site was incorporated into the cDNA of this segment by the introduction of two silent point mutations (T635C and G637C) to differentiate BTV from rBTV. Finally, the recombinant segment encoding the reporter gene was also sequenced in the viral stocks.

### Virus growth kinetics

Multicycle virus growth kinetics were carried out in monolayers of mammalian Vero cells (12-well plate format, 1 × 10^5^ cells/well, triplicates) infected with the indicated viruses at multiplicity of infection (MOI) of 0.01. After 90 min of virus adsorption at 37°C, the inoculum was discarded, cells were washed with DMEM, and 1 mL of growth medium was added. At the indicated times post infection (0, 24, 48, and 72 h), 100 µL of supernatant was removed and stored at −80°C. Virus titers were determined in Vero cells by plaque assay (Plaque-forming units, PFU/mL), as previously described (61, 62, 99, 100). The mean value and standard deviation (SD) were calculated using GraphPad Prism version 8.0.1 (GraphPad Software Inc.).

NLuc activity was quantified at the indicated times (0, 24, 48, and 72 h.p.i.), from tissue culture supernatants of Vero cells (12-well plate format, 1 × 10^5^ cells/well, triplicates) infected as indicated above. For that, 15 µL from the supernatant was used to assess NLuc activity using the Nano-Glo Luciferase Assay kit (Promega) and a FLUOstar Omega microplate reader (BMG Labtech).

### Protein gel electrophoresis and Western blot analysis

Cell extracts from either mock- or virus-infected (MOI 0.01) Vero cells were lysed at 48 h.p.i. in radioimmunoprecipitation assay buffer (Santa Cruz Biotechnology), and proteins were separated by denaturing electrophoresis as previously described (61, 62). Membranes were blocked for 1 h with 5% dried skim milk in PBS containing 0.1% Tween 20 (T-PBS) and incubated overnight at 4°C with specific primary monoclonal or polyclonal antibodies (MAbs or pAbs, respectively): NS2 (mouse MAb 23H6, Eurofins INGENASA, Madrid, Spain), GFP (goat pAb, Rockland), and NLuc (MAb, MAB10026, R&D Systems). A MAb against actin (MAb A1978; Sigma) was used as an internal loading control. Bound primary antibodies were detected with horseradish peroxidase (HRP)-conjugated secondary antibodies against the different species. Proteins were detected by chemoluminescence (Thermo Fisher Scientific) following the manufacturer’s recommendations and photographed using a Bio-Rad ImageStation.

### Confocal microscopy

Vero cells were grown in glass coverslips and non-infected or infected with rBTV-1, rBTV-4, rBTV-8, rBTV-1/Venus, rBTV-4/Venus, or rBTV-8/Venus at a MOI of 0.1. Twenty-four hours after infection, cell monolayers were fixed for 15 min with paraformaldehyde 4%. Fixed cells were blocked with 20% FBS-PBS-0.2% saponine (20% blocking solution) for 60 min at room temperature (RT). Cells were then incubated overnight at 4°C with a mouse hyperimmune serum against NS1 (generated in the laboratory) (61) (1:500) or the NS2 BTV-specific monoclonal antibody (mAb) 23H6 (Eurofins INGENASA, Madrid, Spain) (1:500) diluted in 20% blocking solution. After three serial washing steps with PBS, cells were incubated for 30 min at RT with Alexa Fluor 594 goat conjugated anti-mouse IgG (Invitrogen, German Town, MD, USA) (1:1,000). Coverslips with infected Vero cells were washed three times with PBS and once with PBS-DAPI (1:10,000). Laser scanning confocal microscopy images were acquired with an inverted Zeiss Axiovert LSM 880 microscope. Images were analyzed with Zen 2.0 (Carl Zeiss) and Fiji (NIH) software packages.

### Electron microscopy

Monolayers of mammalian Vero cells (6-well plate format, 1 × 10^5^ cells/well) were infected with rBTV-1 or rBTV-1/Venus at a MOI of 0.1. Twenty-four hours after infection, cell monolayers were fixed 4% PFA and 2% glutaraldehyde (GA) in 0.1 M phosphate buffer, pH 7.4 for 120 min at RT. Post-fixation was carried out with 1% OsO4 and 0.8% K_3_Fe(CN)_6_ in water at 4°C for 1 h. Samples were dehydrated with ethanol and embedded in Epoxy, TAAB 812 Resin (TAAB Laboratories) according to standard procedures. After polymerization, 70 nm ultrathin sections were obtained and stained with uranyl acetate and lead citrate according to standard procedures. Samples were examined in a Jeol 1400Flash microscope operating at 100 kV. Images were recorded with an OneView (Gatan, USA) CMOS digital camera.

To determine the mean diameter of the NS1 tubules formed in rBTV1 and rBTV-1/Venus infections, tubular cross-sections (65 per condition) were analyzed at a magnification of 15,000× using the measurement line tool of Digital Micrograph software (Gatan, USA).

### Stability of reporter-expressing viruses in cultured cells

Vero cells (12-well plate format) were infected (MOI, 0.01) with the indicated viruses (rBTV-1/NLuc, rBTV-1/Venus, rBTV-4/NLuc, rBTV-4/Venus, rBTV-8/NLuc, or rBTV-8/Venus) and incubated until a 70% CPE was observed. Tissue culture supernatants were then harvested and 1/10 of the collected medium was used for the infection of fresh Vero cells for a total of 5 passages. During each viral passage, cultured supernatants were collected and plaque assays were carried out as described above. Venus-expressing plaques were visualized directly using a Zeiss Axio fluorescence microscope (Zeiss). To determine the stability of NLuc-expressing viruses, plaques (*n* = 30) were used to infect fresh Vero cells (96-well plate format, 2.5 × 10^4^ cells/well) and NLuc activity from tissue culture supernatants was determined at 48 h.p.i., as indicated above. The graph shows the % of plaques that express NLuc compared to the total number of plaques examined in each passage. About 30 plaques per passage were evaluated for each virus.

### Pathogenicity and vaccine studies

Type I interferon receptor defective mice (IFNAR(−/−)) on a 129 Sv/Ev background were bred in the animal care facility of the Department of Animal reproduction at INIA and housed under pathogen-free conditions at the biosafety level 3 (BSL3) animal facilities in the Animal Health Research Center (CISA-INIA/CSIC), Madrid (Spain). Animal experimental protocols were approved by the Ethical Review Committee at the CISA-INIA/CSIC and Comunidad de Madrid (Permit number: PROEX 060.7/21), in strict accordance with EU guidelines 2010/63/UE about the protection of animals used for experimentation and the Spanish Animal Welfare Act 32/2007.

For pathogenicity studies, 8-week-old mice (*n* = 5 per group) were subcutaneously inoculated with the indicated PFUs of rBTV-1, rBTV-1/NLuc, rBTV-4, rBTV-4/NLuc, rBTV-8, or rBTV-8/NLuc. Then, animals were monitored daily during 2 weeks for clinical symptoms (such as malaise, respiratory distress, and lack of movement), and mortality.

For vaccine studies, a group (*n* = 10) of 12-week-old mice was immunized following a homologous prime-boost regime consisting of two subcutaneous doses of 5.58 µg in 100 µL per mouse of a commercial inactivated vaccine against BTV-8 (Bluevac BTV, CZ VETERINARIA S.A.) administered 4 weeks apart. A group (*n* = 10) of 12-week-old mice was mock-immunized (control). Then, animals were subcutaneously challenged with a lethal dose (1,000 PFU) of rBTV-8/NLuc 4 weeks after boost dose.

To evaluate RNAemia and NLuc activity, at 3, 5, 7, 10, and 15 d.p.i., blood samples from the submandibular plexus were collected in tubes with ethylenediamine tetraacteic acid (EDTA) as anti-coagulant. RNA was extracted from 50 µL of blood using TRIzol (Invitrogen) following the manufacturer’s instructions. RNAemia was determined in duplicate by real-time RT-qPCR specific for BTV segment 5, as described by Toussaint et al. (101). Briefly, RT-qPCR was carried out with the AgPath-ID One-Step RT-PCR Reagents (Thermo Fisher Scientific) using 400 nM of each primer (GGCAACYACCAAACATGGA and AAAGTYCTCGTGGCATTWGC) (Sigma AldrichA), 200 nM of the Taqman probe conjugated to FAM at the 5-end and to TAMRA at the 3-end (FAMCYCCACTGATRTTGTATTTTCTCAA-TAMRA) (Sigma Aldrich), and 2 µL of RNA extracted from 50 µL of blood. Cycling conditions were as follows: 1 cycle at 48°C for 25 min, 1 cycle of 95°C for 15 min, 45 cycles with 15 s at 95°C, and 1 min at 61°C. RT-qPCR was performed on an Illumina ECOTM thermal cycler (ECOTM Real-Time PCR System, Illumina). Only Ct values lower than 38 were considered indicative of RNAemia (positive), according to the cut-off established by Toussaint et al. (101).

To assess the presence of NLuc in blood samples, plasma from 50 µL of whole blood was obtained and the NLuc activity in 15 µL of sample was measured using the Nano-Glo Luciferase Assay kit (Promega) and a FLUOstar Omega microplate reader (BMG Labtech). Analysis of the correlation between NLuc activity and RNAemia levels was done using GraphPad PRISM 8 (GraphPad Software, San Diego, CA) by calculation of Spearman’s rank correlation coefficient, and their relationships were modeled by linear regression.

### *In vivo* bioluminescence of rBTV-8/NLuc

*In vivo* bioluminescence imaging of whole mice was performed with an IVIS Spectrum multispectral imaging instrument (PerkinElmer, Inc.). The mice were lightly anesthetized with isoflurane, and Nano-Glo luciferase substrate (Promega) was diluted 1:10 in PBS and injected retro-orbitally in a final volume of 100 µL. The mice were immediately imaged, and bioluminescence data acquisition and analysis were performed using Living Image software (v4.5; PerkinElmer). Flux measurements were acquired from the region of interest. The scale used for the data is included in each specific figure. The Brain, spleen, lungs, heart, kidney, liver, and testis/ovaries were surgically extracted and washed with PBS, and substrate was added directly to the collected organs. Images were acquired and analyzed with Living Image (v4.5) software to determine the radiant efficiency of the regions of interest.

### Statistical analysis

Microsoft Excel (Microsoft Corporation) and GraphPad Prism version 8.0.1 (GraphPad Software, San Diego, CA, USA) software were used to analyze the data. Microsoft Excel was necessary to perform some of the calculations and to visualize the raw data. GraphPad Prism software was used to perform statistical analysis on all data. Growth kinetics were analyzed using multiple *t* test analysis using the Holm-Šídák method. Statistical differences between survival curves were analyzed using log-rank test. Comparisons of mean responses of RNAemia levels and luminescent signals between groups were conducted by multiple *t* test analysis using the Holm-Šídák method. Correlation between RNAemia and luminescent activity was analyzed by nonparametric Spearman’s rank order correlation coefficient. A *P* value lower than 0.05 was considered significant in all cases. Size of virus lysis plaques was compared by unpaired *t*-test.
